# Cryptogenic Stroke Complicated by Infective Endocarditis: Exploring the Multidisciplinary Interplay

**DOI:** 10.7759/cureus.58945

**Published:** 2024-04-24

**Authors:** Ameer Khan, Faisal Hanif, Amina Arif, Fawzhiah T Haque, Sean Donnelly

**Affiliations:** 1 Cardiology, Tameside General Hospital, Ashton-under-Lyne, GBR; 2 Cardiology, University of Manchester, Manchester, GBR

**Keywords:** streptococcus mitis, mental wellbeing, infective discitis, native mitral valve, infective endocarditis

## Abstract

Infective endocarditis (IE) poses a significant clinical challenge due to its non-specific symptoms and variety of complications. Complications can include ischaemic stroke, valve dysfunction, discitis, and osteomyelitis, highlighting the complexity of IE management. We present a case of a male in his 40s, admitted with an ischaemic stroke, eventually being found to have underlying IE with a plethora of complications. This case highlights the importance of collaboration among specialists to form a multidisciplinary team, which is essential for the effective delivery of care. Furthermore, there is a critical need to explore the psychological impact of IE on patient outcomes, advocating for a holistic approach that considers psychological well-being alongside medical management. Future research should address these underexplored facets to improve patient care and outcomes in IE.

## Introduction

Infective endocarditis (IE), characterised by inflammation of the inner cardiac membrane, is associated with an alarmingly high mortality rate of over 25% [1]. Symptoms are non-specific and include fever, lethargy, and malaise. IE is associated with a variety of complications that further hinder clinical outcomes. One of the most severe complications of IE is ischaemic stroke, which occurs as a result of intracardiac vegetation embolism, often in multiple sites in the brain [2]. Valvular dysfunction also occurs as a result of intracardiac vegetation, whilst musculoskeletal issues can occur due to the spread of infection to bone and tissue [2,3].

The complexity of diagnosing and treating IE in patients, and managing a variety of complications, highlights the importance of a multidisciplinary approach to the disease. This case report presents a male in his 40s who was admitted to our acute stroke unit with neurological deficits suggestive of ischaemic stroke. Further evaluation revealed cerebral haemorrhages, mitral and aortic valve dysfunction, and concurrent discitis and osteomyelitis. The management of such complex cases necessitated collaboration among specialists from neurosurgery, orthopaedics, cardiology, microbiology, and infectious diseases.

## Case presentation

A male in his 40s presented to the hyperacute stroke unit with left-sided weakness, expressive dysphasia, right-sided facial droop, and slurring of speech. He was graded a modified Rankin Scale 4. Admission CT imaging of the brain showed signs of an ischaemic stroke. Blood results showed a normocytic anaemia of unclear origin. The patient was noted to be in the thrombolysis window. Prior to treating the infarct with thrombolysis, two units of blood were transfused. An admission ECG was shown to be in sinus rhythm with no other abnormalities.

**Figure 1:**
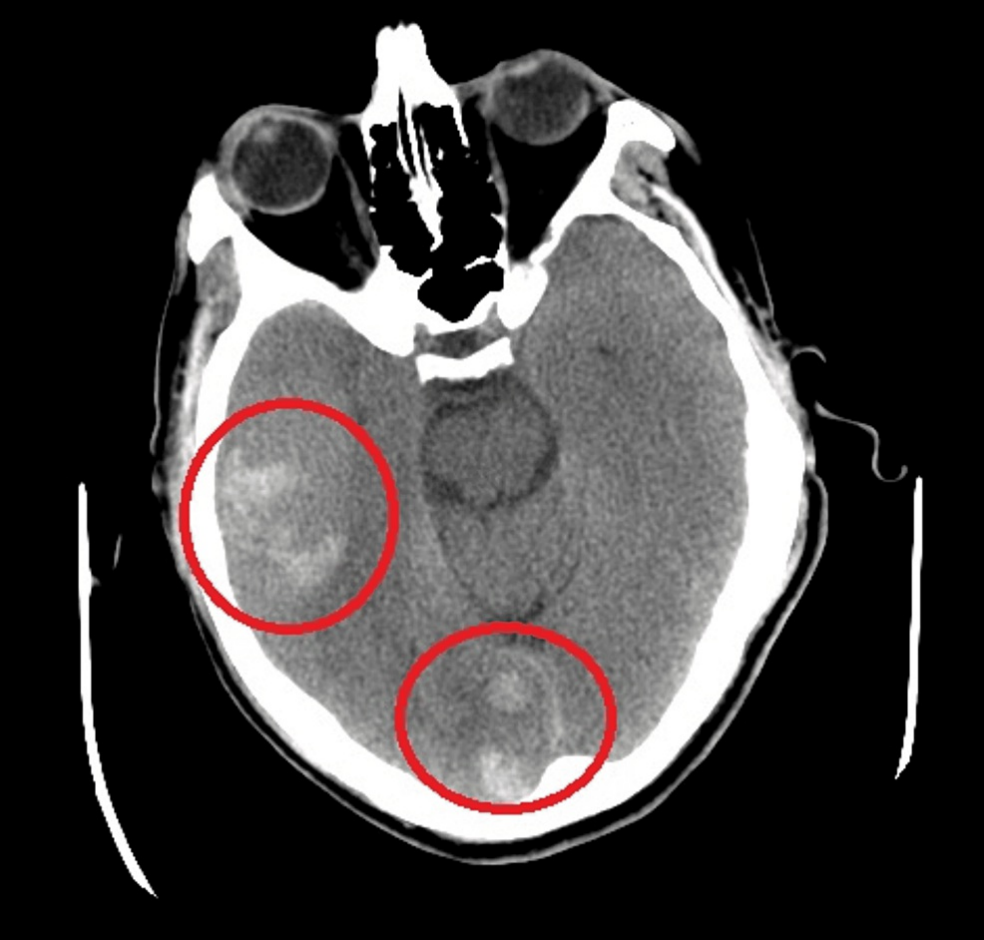
CT of the brain showing multifocal, right hemispheric, and right cerebellar haemorrhages with associated signs of a subarachnoid haemorrhage. Red circles show areas of haemorrhage.

Repeat CT scan one hour post-alteplase showed multifocal, right hemispheric, and right cerebellar haemorrhages with associated subarachnoid haemorrhage (Figure 1).

Subsequent neurosurgical review deemed no further intervention was needed and for monitoring to continue with periodic neurological observations. Auscultation on admission revealed a pansystolic murmur, and blood tests showed elevated CRP levels with no other apparent signs of infection.

The patient denied a history of smoking, alcohol, or any illicit drug use. No allergies were noted. Past medical history included benign positional paroxysmal vertigo. His only medication of note was amitriptyline for his recent back pain.

**Figure 2:**
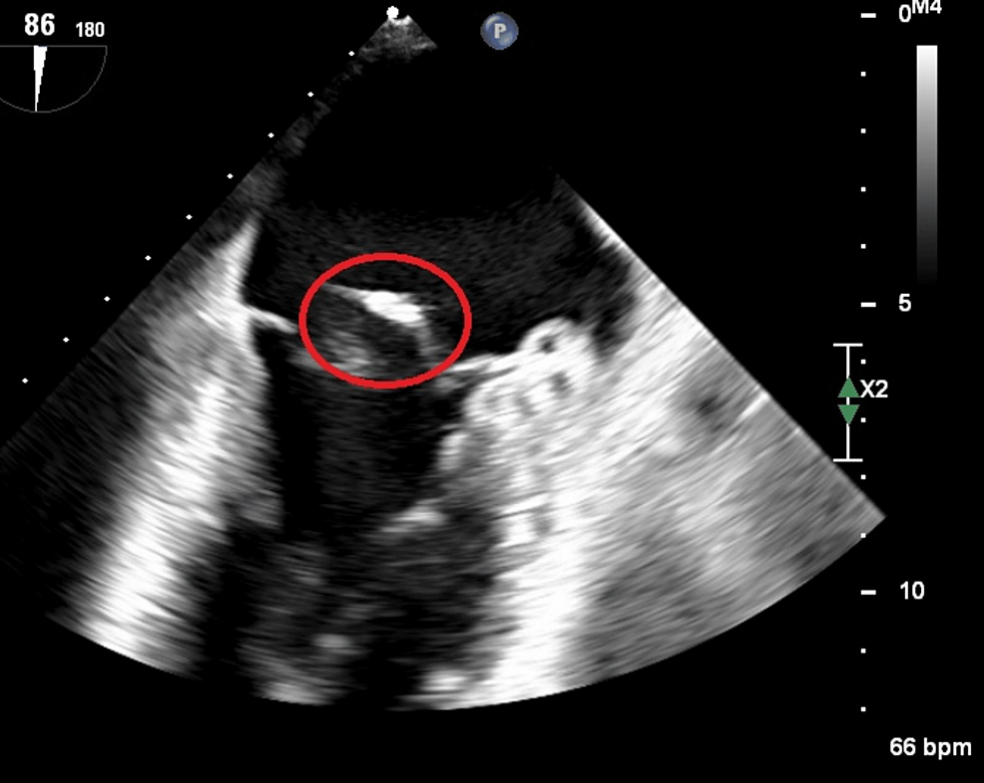
Transoesophageal echocardiographic image showing vegetation on mitral valve leaflet. The red circle demarcates vegetation.

Blood cultures returned positive for *Streptococcus mitis*, and intravenous benzylpenicillin was initiated. Transthoracic echocardiography showed a persevered ejection fraction at 55% with vegetation on the posterior mitral valve leaflet and evidence of severe mitral regurgitation, with an anterior directed jet transoesophageal echocardiography corroborated earlier echo findings of vegetation on the posterior mitral valve leaflet, and also revealed mild aortic regurgitation (Figure 2).

The patient was listed for a peripherally inserted central catheter (PICC) line to receive long-term antibiotics.

**Figure 3:**
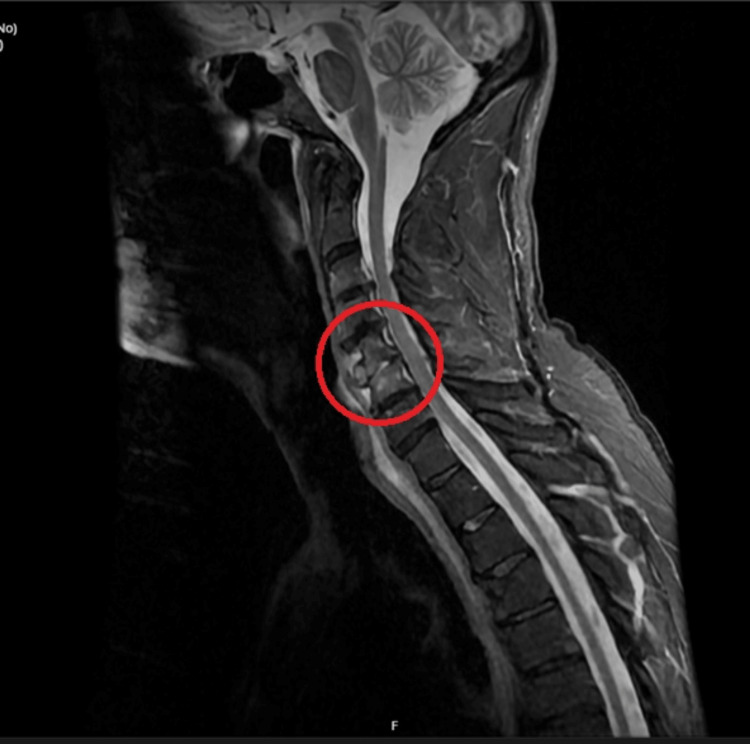
MRI showing discitis at the level of vertebrae C4 & C5. The red circle demarcates the area of discitis.

The patient also stated that he had been experiencing back pain for 10 weeks, which he felt could have stemmed from a fall he had sustained a few weeks prior. Though neurological examination elicited no sensory or motor deficit, there was marked tenderness over the cervical and thoracic vertebrae. MRI of the spine showed discitis at the vertebral level of C4 and C5. An MRI scan with contrast was requested on the advice of the neurosurgical spinal team and showed osteomyelitis (Figure 3).

It was subsequently decided from multidisciplinary team (MDT) meetings of both the infective endocarditis and spinal surgeons that continuation of antibiotics was sufficient at this stage.

During his acute admission, the patient developed clinical signs of depression and a withdrawal in his behaviour was noted. Acute mental health input was sought and a recommendation was given for an outpatient referral and management advice from primary care.

Following a six-week course of intravenous antibiotics, blood cultures were negative, and it was agreed following evaluation by the microbiologists, neurosurgeons, and other members of the MDT that the patient could be stepped down to oral antibiotic therapy. Before discharge, the patient mentioned that three months prior to his admission, he had sought emergency dental treatment whilst abroad on holiday.

A cervical collar was given to the patient to be used upon mobilisation, as advised by the orthopaedic and spinal surgeons. From a cardiology perspective, the patient would be followed up closely as an outpatient to ensure that his heart function remained preserved. Cardiothoracic surgeons were to follow up the patient for the severe mitral valve regurgitation, as the patient did not have any class one indications for an immediate surgical replacement of the valve.

## Discussion

IE is a multifaceted challenge in clinical practice with a variety of presentations. Recognition of IE and prompt treatment with antibacterial agents are associated with reduced in-hospital mortality and morbidity [4].

However, patients are usually referred to specialised centres only after serious complications have developed. Our patient presented initially with symptoms of stroke without fever or known valvular disease but was still found to have IE. Further tests revealed mitral and aortic dysfunction, and discitis and osteomyelitis, all likely to have occurred as complications of IE. The European Society of Cardiology (ESC) guidelines recommend the use of MDTs in the care of patients with IE and have emerged as an effective means of attaining faster diagnosis and treatment [5].

A recent meta-analysis showed that the establishment of an MDT was linked with improved short-term mortality, whilst another study showed that the implementation of an MDT was linked to a reduction in mortality by 50%, lower rates of renal and multi-organ failure, and fewer deaths by embolic events [6,7]. Improvements were seen in a separate study with MDTs by overall in-hospital and three-year mortality rates, whilst Sanchez et al. found that MDT establishment led to improved detection of IE and more early elective surgeries for patients with IE, leading to a 13% reduction in overall in-hospital mortality [8,9].

Our patient developed osteomyelitis and discitis, warranting input from the neurosurgical orthopaedic, microbiology, cardiology, and infectious disease specialists. Medical management of IE is with two to six weeks of high-dosage antibiotics and is linked with improved outcomes when given promptly [5]. It is aimed at eradicating the infection and preventing relapses. It can also be used to address complications, as in the case of our patient where benzylpenicillin for IE was also used to target the patient’s discitis and osteomyelitis [5]. Late embolic episodes from IE have been known to cause cases of discitis [10]. Studies in the past have gone on to show that the symptoms of IE would appear after the back pain [11].

In investigating IE, establishing a cause is important to help direct management and prevent any further reoccurrences. *Streptococcus mitis* is one of the most common inhabitants of the oral cavity and has been shown to be a causative organism for IE [12]. Patient education on maintaining safe oral hygiene practices is crucial in the long-term management of such patients.

In addition to antibacterial therapy, the patient was to undergo close surveillance as an outpatient, in the instance he developed a class I or II indication for mitral valve replacement surgery [5]. Surgery is indicated in the event of acute heart failure, extensive infection with localised complications, and recurrent arterial embolisation, which has been shown to be necessary in 50% of patients with IE [5-13].

While physical manifestations of IE have been widely explored, much less work has been done on analysing the psychological impact of the disease. Long hospital stays are known to increase anxiety and depression symptoms in the general population, and IE patients often require longer hospital stays than other patients [14]. Studies have shown lower levels of quality of life (QoL) in IE patients post-treatment compared to healthy controls. Verhagen et al. found that over 10% of IE patients developed post-traumatic stress disorder, along with symptoms of anxiety and depression, and difficulties in functioning at work and in social activities [15]. Even less is known about specific factors that contribute to such psychological issues. Bundgaard et al. showed that IE patients receiving partial-oral outpatient treatment reported improvements in anxiety and depression at six months more than conventionally treated intravenous patients, though this was not significant [16]. One study found that optimal antibacterial therapy as well as early surgical intervention were associated with improved QoL [17]. Though the impact of psychological hindrance on mortality is not known specifically for patients with IE, a positive association has been found in other cardiac populations, but whether this translates to IE is unknown [18].

Psychological deterioration represents an underappreciated facet within the overall complexity of infective endocarditis, and work is needed to ascertain the impact of QoL on mortality in IE, and to determine the biggest contributor(s) within the development/diagnosis/treatment of IE to poor QoL.

## Conclusions

IE management is characterised by its intricate and varied components. This case highlights the necessity of MDTs in diagnosing and treating patients with IE, especially where several multi-system complications occur. As physicians, patient-centred care is vital to ensure we meet the individual needs of patients. The need for further research on the psychological impact of IE is evident, as psychological well-being remains an under-explored aspect of the disease. A holistic approach that includes addressing the psychological well-being of patients with IE may be beneficial in improving patient outcomes.
